# Impact of local CNP/GC-B system in growth plates on endochondral bone growth

**DOI:** 10.1186/2050-6511-14-S1-P48

**Published:** 2013-08-29

**Authors:** Kazumasa Nakao, Akihiro Yasoda, Kenji Osawa, Toshihito Fujii, Eri Kondo, Noriaki Koyama, Naotetsu Kanamoto, Masako Miura, Koichiro Kuwahara, Haruhiko Akiyama, Kazuhisa Bessho, Kazuwa Nakao

**Affiliations:** 1 Department of Oral and Maxillofacial Surgery, Kyoto, Japan; 2 Department of Medicine and Clinical Science, Kyoto, Japan; 3 Department of Orthopedic Surgery Kyoto University Graduate School of Medicine, Kyoto, Japan

## Background

C-type natriuretic peptide (CNP), which exerts its biological actions by binding a subtype of receptor, guanylyl cyclase-B (GC-B), is implicated in the endochondral bone growth and the gene disruption of CNP or GC-B results in marked dwarfism due to the defect of the endochondral bone formation in both animals and humans. Meanwhile, the physiological role of the CNP/GC-B system in the growth plate cartilage is still unclear, because the CNP/GC-B system is widely distributed not only in the growth plate of the bone but also in the central and peripheral nervous system, gonad, vascular endothelial cells, macrophages and cardiac fibroblasts.

## Results

In order to further elucidate the physiological role of the local CNP/GC-B system in the growth plate cartilage, we have performed targeted depletion of CNP or GC-B gene in the growth plate cartilage, using the Cre-loxP system. The growth of bones formed through the endochondral ossification was severely impaired in cartilage-specific CNP knockout mice, resulting in a prominent short-stature phenotype. The severity of impairment of endochondral bone growth observed in cartilage-specific CNP knockout mice was almost the same as in the systemic CNP knockout mice. The bone phenotype of cartilage specific GC-B knockout mice was essentially the same as that of CNP knockout mice, however, the shortening of bones was severer in cartilage specific GC-B knockout mice than that in cartilage specific CNP knockout mice. Histological examination revealed that the hypertrophic chondrocyte layer of the growth plate was drastically reduced and the thickness of the proliferative chondrocyte layer, along with the proliferation of chondrocytes in that region, was moderately reduced in both cartilage specific knockouts. On pulverized feed, the survival rate of cartilage specific CNP and GC-B knockout mice was comparable to that of systemic CNP knockout mice.

## Conclusion

These results demonstrate that the local CNP/GC-B system in growth plates is responsible for the physiological endochondral bone growth.

